# Hemolytic activity of venom from crown-of-thorns starfish *Acanthaster planci* spines

**DOI:** 10.1186/1678-9199-19-22

**Published:** 2013-09-24

**Authors:** Chi-Chiu Lee, Wann-Sheng Tsai, Hernyi Justin Hsieh, Deng-Fwu Hwang

**Affiliations:** 1 Department of Food Science and Center of Excellence for Marine Bioenvironment and Biotechnology, National Taiwan Ocean University, Taiwan, ROC; 2 Penghu Marine Biology Research Center, Fisheries Research Institute, Council of Agriculture, Taiwan, Magong, Penghu, ROC; 3 Department of Health and Nutrition Biotechnology, Asia University, Taiwan, ROC

**Keywords:** Crown-of-thorns starfish, Spine, Venoms, Hemolysis

## Abstract

**Background:**

The crown-of-thorns starfish *Acanthaster planci* is a venomous species from Taiwan whose venom provokes strong hemolytic activity. To understand the hemolytic properties of *A. planci* venom, samples were collected from *A. planci* spines in the Penghu Islands, dialyzed with distilled water, and lyophilized into *A. planci* spine venom (ASV) powder.

**Results:**

Both crude venom and ASV cause 50% hemolysis at a concentration of 20 μg/mL. The highest hemolytic activity of ASV was measured at pH 7.0-7.4; ASV-dependent hemolysis was sharply reduced when the pH was lower than 3 or greater than 8. There was almost no hemolytic activity when the Cu^2+^ concentration was increased to 10 mM. Furthermore, incubation at 100°C for 30 to 60 minutes sharply decreased the hemolytic activity of ASV. After treatment with the protease α-chymotrypsin, the glycoside hydrolase cellulase, and the membrane component cholesterin, the hemolytic activity of ASV was significantly inhibited.

**Conclusions:**

The results of this study provide fundamental information about *A. planci* spine venom. The hemolytic activity was affected by pH, temperature, metal ions, EDTA, cholesterin, proteases, and glycoside hydrolases. ASV hemolysis was inhibited by Cu^2+^, cholesterin, α-chymotrypsin, and cellulose, factors that might prevent the hemolytic activity of venom and provide the medical treatment for sting.

## Background

The crown-of-thorns starfish *Acanthaster planci* is an Echinoderm from the class Asteroidea, the order Valvatida, and the family Acanthasteridae. In the past, *A. planci* was rarely reported in Taiwan. However, in recent years, it has bloomed in coastal areas of Penghu, Taiwan. The crown-of-thorns starfish destroys coral reefs and has been involved in significant events, such as abnormal outbreaks of this species. Consequently, coral destruction and population decreases of organisms that depend on coral for food have occurred worldwide. In addition to being a known coral reef destroyer, *A. planci* is a venomous starfish. Skin injuries due to the crown-of-thorns starfish have been observed and are considered to be a serious medical issue [1,2].

*A. planci* venomous spines can deliver stings that provoke various pathological symptoms, such as pain and protracted vomiting, and signals like erythema and swelling. Although some of the skin surface lesions of spine-sting patients show improvement, pain and subcutaneous indurations persist in most of the lesions. Radiographies of victims usually show several remaining spines. Thus, surgical excision to remove the spines is normally required, after which the symptoms tend to improve.

Previous studies showed that the crude toxin extracted from the spines exhibits diverse biological activities, including hemolysis, mouse lethality, edema formation, phospholipase A_2_ (PLA_2_) activity, and anticoagulant activity [3-5]. A hemolytic activity assay was used as an initial approach to characterize the starfish venom because it is a rapid, easily reproducible and quantifiable method [6]. Although previous research found that despite the hemolytic activity presented by proteinaceous venom from the crown-of-thorns starfish, such activity has not been widely investigated; a situation that inhibits the proper care of patients after envenomation accidents. Some marine creatures like the jellyfish, cone snail, stone fish and crown-of-thorns starfish possess hemolytic venom. Several studies reporting the factors affecting the hemolytic activity of jellyfish venom have been studied to determine effective treatment for envenomation by jellyfishes [4-7].

The present study aimed to provide a preliminary description of the protein features of *A. planci* venom by investigating its hemolytic activity and related stability properties. This study represents a basis for further research on the biological activities and pharmacological uses of *A. planci* venom.

## Methods

### Preparation of starfishes

Ten *A. planci* specimens with body weights of 400-500 g were captured during 2010 and 2011 along the coast of Penghu, Taiwan. After capture, the specimens were immediately frozen, transported to the laboratory, and stored at –20°C until use.

### Venom extraction

Venom collected from 100 g of spines from the frozen specimens of *A. planci* was homogenized and extracted two times using two volumes of 0.01 M phosphate buffered saline (PBS) (KH_2_PO_4_ 1.8 mM, Na_2_HPO_4_ 10 mM, NaCl 0.15 M and KCl 2.7 mM) (pH 7.4). The venom samples were then centrifuged at 4°C for 20 minutes at 14,000 g. The supernatant was collected as crude venom from the *A. planci* spines, dialyzed against distilled water using dialysis membranes (UC30-32-100, Sanko Junyaku Co., Japan; separation MW: 12,000 to approximately 14,000 Da) to remove the salt, and lyophilized into *A. planci* spine venom (ASV) powder. The lyophilized powder from 100 g of spines constituted about 1.12 g. Then, 0.01 g of ASV powder was dissolved in 1 mL of 0.01 M PBS buffer with a pH of 7.4 to make a stock solution that was further diluted into different concentrations of ASV test solution.

The protein concentration of the ASV powder was determined through the method described by Bradford [8] and compared with bovine serum albumin (BSA) protein concentration standards.

### Hemolytic activity assay

In brief, 0.5 mL of a 0.05% suspension of rat erythrocytes in a 0.01 M PBS buffer with a pH of 7.4 was mixed with 0.5 mL of different concentrations of ASV (5 to 40 μg/mL) and incubated at 37°C for 30 minutes. Absorbance of 0.01 M PBS buffer mixed with rat erythrocytes was used as control, and absorbance of saponin (*Quillaja bark*) (15 μg/mL) mixed with rat erythrocytes indicated maximal lysis. The hemolysis percentage was determined at the end of the assay using the following equation:

$$\begin{array}{l} \;\mathrm{Hemolysis}\left(\%\right)=\frac{\left(\mathrm{Abs}.\mathrm{of}\;\mathrm{sample}-\mathrm{Abs}.\mathrm{of}\;\mathrm{control}\right)}{\left(\mathrm{Abs}.\mathrm{of}\;\mathrm{maximal}\;\mathrm{lysis}–\mathrm{Abs}.\mathrm{of}\;\mathrm{control}\right)}\times 100\\ \; \end{array}$$

HU_50_ was defined as the amount of ASV required to cause 50% lysis.

### Effect of pH on the hemolytic activity of ASV

To assay the effect of pH on the hemolytic activity, ASV powder was dissolved in a 0.01 M PBS buffer (pH 7.4) and adjusted with 0.1 N HCl and 0.1 N NaOH to create samples of various pH values (2, 3, 4, 5, 6, 7, 8, 9, and 10). The hemolysis of these samples was tested after a 30-minute incubation.

### Effect of heat on the hemolytic activity of ASV

To test the effect of heat on the hemolytic activity of *A. planci* venom, the ASV solution was assessed for hemolysis after incubation at different temperatures (40, 60, 80, and 100°C) for 15 minutes, 30 minutes, and 60 minutes.

### Effect of different metal ions and EDTA on the hemolytic activity of ASV

The effect of six different metal ions (Na^+^, K^+^, Ca^2+^, Mg^2+^, Cu^2+^, Fe^3+^) and EDTA on the hemolytic activity of ASV were assayed by adding their chloride salts (NaCl, KCl, CaCl_2_, MgCl_2_, CuCl_2_, FeCl_3_) to the venom. The hemolytic activity was assayed as described above. The metal ions and EDTA were tested at concentrations of 0.001, 0.01, 0.1, 1, and 10 mM.

### Effect of cholesterin on the hemolytic activity of ASV

To clarify the ASV target on the erythrocyte membrane, the effect of membrane lipids, such as cholesterin, on the hemolytic activity of venom was assayed. Cholesterin was prepared as a dispersion using homogenization in PBS buffer. The venom of *A. planci* was incubated with cholesterin at 4°C for 30 minutes, after which the hemolytic activity was assayed.

### Effect of proteases on the hemolytic activity of ASV

The effect of proteases (pepsin, α-chymotrypsin, and trypsin) on the hemolytic activity of the venom were tested by incubating aliquots of the venom mixed with protease concentrations of 5, 10, and 50 U/mL for 30 minutes at 37°C. Treated venom samples were assayed on erythrocytes as described previously.

### Effect of glycoside Hydrolases on the hemolytic activity of ASV

The effects of glycoside hydrolases (α-amylase, xylanase and cellulase) on the hemolytic activity of the venom were tested by incubating aliquots of the venom mixed with glycoside hydrolase concentrations of 5, 10, 20, and 40 U/mL for 30 minutes at 37°C. Treated venom samples were assayed upon erythrocytes as described above.

### Statistical analysis

All data were expressed as the mean ± SD of three parallel measurements, and analyzed using the Student’s *t* test. All tests were considered statistically significant at p < 0.05.

### Ethics committee approval

The present study was approved by the National Taiwan Ocean University Experimental Animal Center (Keelung, Taiwan, ROC).

## Results

### Hemolytic activity assay

**Figure 1:**
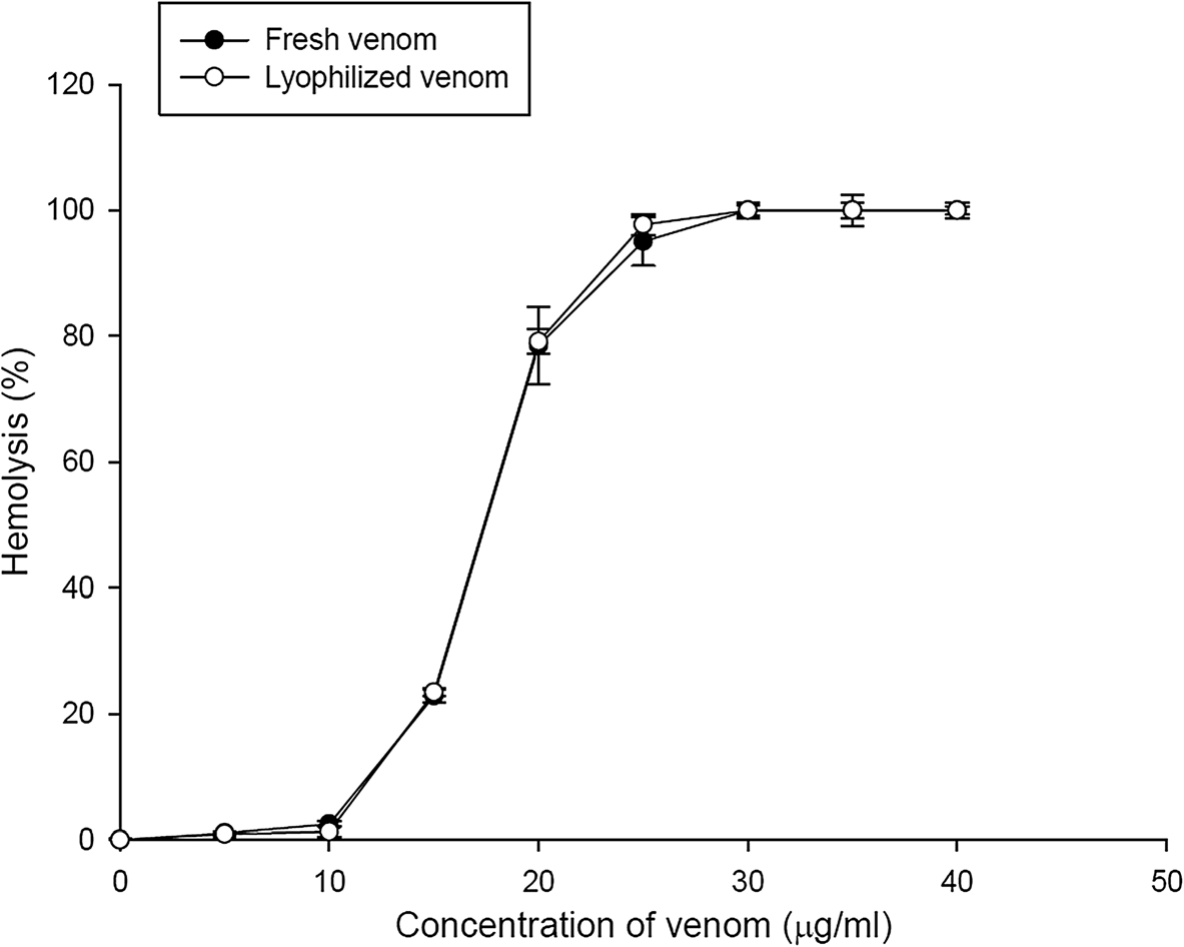
**Dose-response curve of the hemolytic activity of ASV.** The ASV hemolysis was assayed using 0.5% rat erythrocyte suspension. All the results are expressed as the mean ± SD (n = 3).

The protein concentration of the ASV powder was determined by the method of Bradford and compared with BSA protein concentration standards. The result showed that 1 mg ASV powder dissolved in 1 mL PBS buffer contained about 0.25 μg of proteins. Figure 1 shows a dose-response curve of the hemolytic activity of *A. planci* venom. The hemolytic activity of ASV was concentration-dependent. Since the HU_50_ value of fresh venom and ASV powder is approximately 18 μg/mL, the hemolytic activity was assayed utilizing 20 μg/mL.

### Effect of pH on the hemolytic activity of ASV

**Figure 2:**
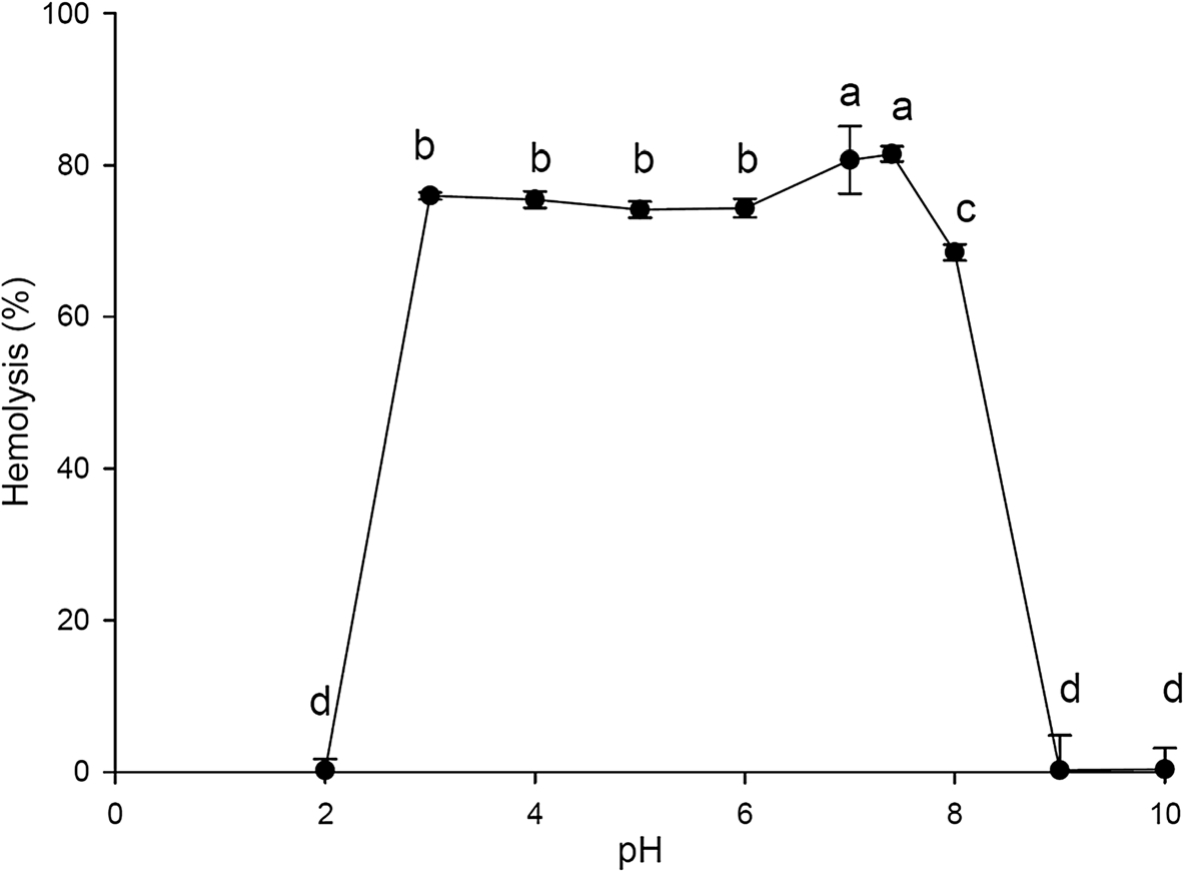
**Effect of pH on the hemolytic activity of the ASV solution (20 μg/mL).** Values **a**, **b**, **c**, **d** differ significantly at *p* < 0.05 (n = 3).

Figure 2 presents the effect of pH on the hemolytic activity of ASV. The hemolytic potency of the venom was reduced in an alkaline environment (pH 8), and in an extremely acidic environment (pH 2.0). The highest percentage (approximately 80%) of ASV hemolysis occurred between pH 7.0 and 7.4. When the pH value was increased above 8.0, a significant decrease in the hemolytic activity of ASV was observed. ASV showed almost no hemolytic activity at pH 9.0, pH 10.0 or pH 2.0.

### Effect of heat on the hemolytic activity of ASV

**Figure 3:**
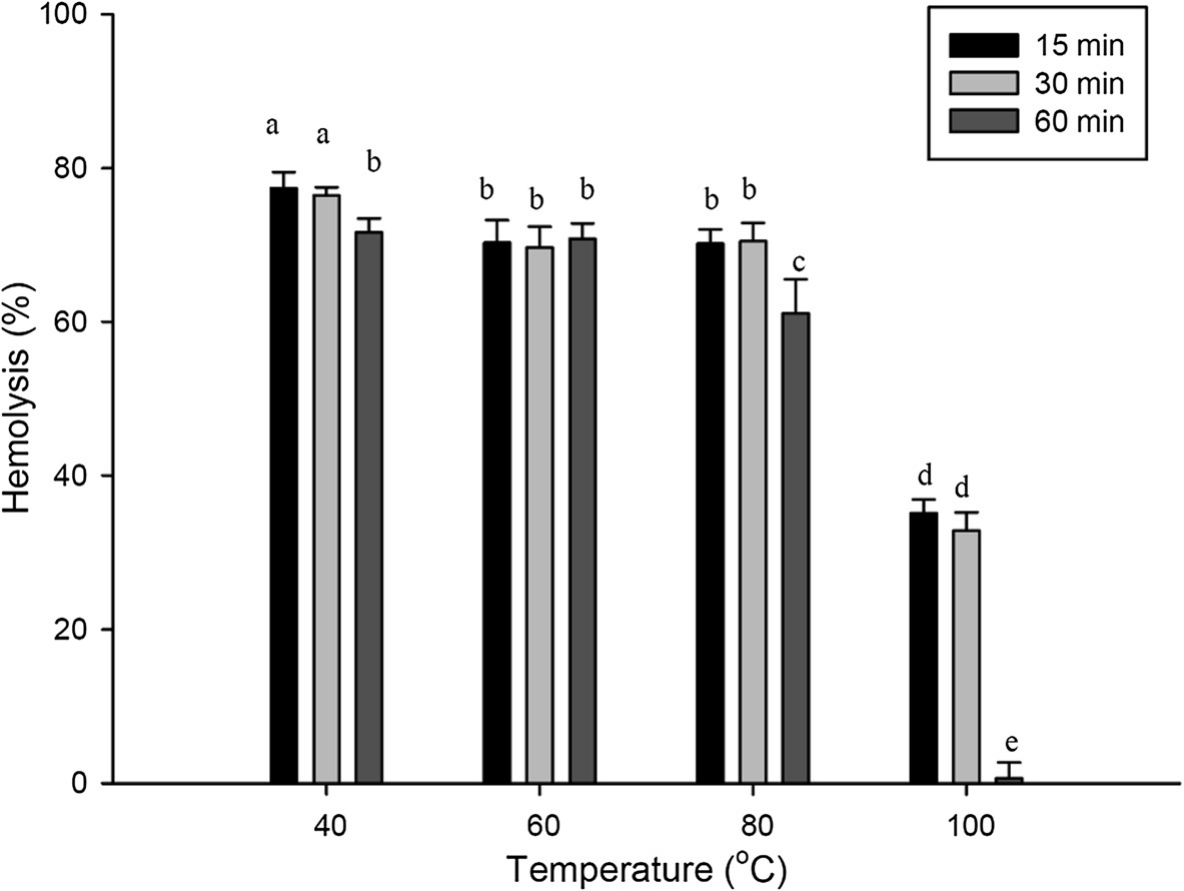
**Effect of temperature (heating for 15, 30, and 60 minutes) on the hemolytic activity of the ASV solution (20 μg/mL).** Values **a**, **b**, **c**, **d**, **e** differ significantly at *p* < 0.05 (n = 3).

The effect of heat on the hemolytic activity of ASV, displayed in Figure 3, shows that ASV presents heat stability. A slight decrease in the hemolytic activity was observed when samples were incubated at 80°C for one hour. The hemolytic activity of ASV was reduced to 40% when samples were incubated at 100°C for 15 or 30 minutes. In particular, the hemolytic activity of ASV was sharply reduced after incubation at 100°C for one hour.

### Effect of different metal ions and EDTA

Table 1 shows the hemolytic activity after treatment with six different metal ions, namely Na^+^, K^+^, Ca^2+^, Mg^2+^, Cu^2+^, and Fe^3+^. Group IA alkali earth metals (Na^+^ and K^+^) and IIA alkali earth metals (Ca^2+^ and Mg^2+^) did not significantly inhibit the hemolytic activity. However, after incubating ASV with 10 mM Ca^2+^, the percentage of hemolysis increased from 78.21 to 99.53%, indicating that Ca^2+^ may improve the hemolytic potency of ASV. Fe^3+^ reduced the hemolytic activity of ASV by approximately 10% only at the high concentration of 10 mM. Only Cu^2+^ had a significant inhibitory effect on the hemolytic activity of ASV. There was almost no hemolytic activity when ASV was mixed with a 10 mM concentration of Cu^2+^.

**Table 1:** Effect of six metal ions on the hemolytic activity of the ASV solution (20 μg/mL)

**Concentration (mM)**	**Hemolysis (%)**					
	**Na**^**+**^	**K**^**+**^	**Ca**^**2+**^	**Mg**^**2+**^	**Cu**^**2+**^	**Fe**^**3+**^
**0.001**	77.01 ± 2.23^a^	77.05 ± 2.71^a^	78.21 ± 0.92^a^	79.17 ± 1.08^a^	79.13 ± 2.53^a^	79.62 ± 2.66^a^
**0.01**	74.59 ± 0.81^a^	75.89 ± 0.72^a^	79.23 ± 1.13^a^	78.95 ± 0.64^a^	78.54 ± 0.67^ab^	78.68 ± 4.06^b^
**0.1**	75.35 ± 0.57^a^	76.64 ± 1.47^a^	89.11 ± 0.77^b^	77.41 ± 0.62^a^	76.22 ± 1.24^bc^	76.47 ± 3.93^b^
**1**	75.16 ± 3.82^a^	75.97 ± 3.20^a^	89.46 ± 0.59^b^	75.36 ± 1.85^a^	71.58 ± 0.38^c^	76.79 ± 3.12^b^
**10**	76.42 ± 0.83^a^	77.31 ± 1.22^a^	99.53 ± 2.61^c^	77.57 ± 1.41^a^	8.03 ± 1.56^d^	69.64 ± 0.92^c^

^a^, ^b^, ^c^, ^d^: Values differ significantly at *p* < 0.05 (n = 3).

**Figure 4:**
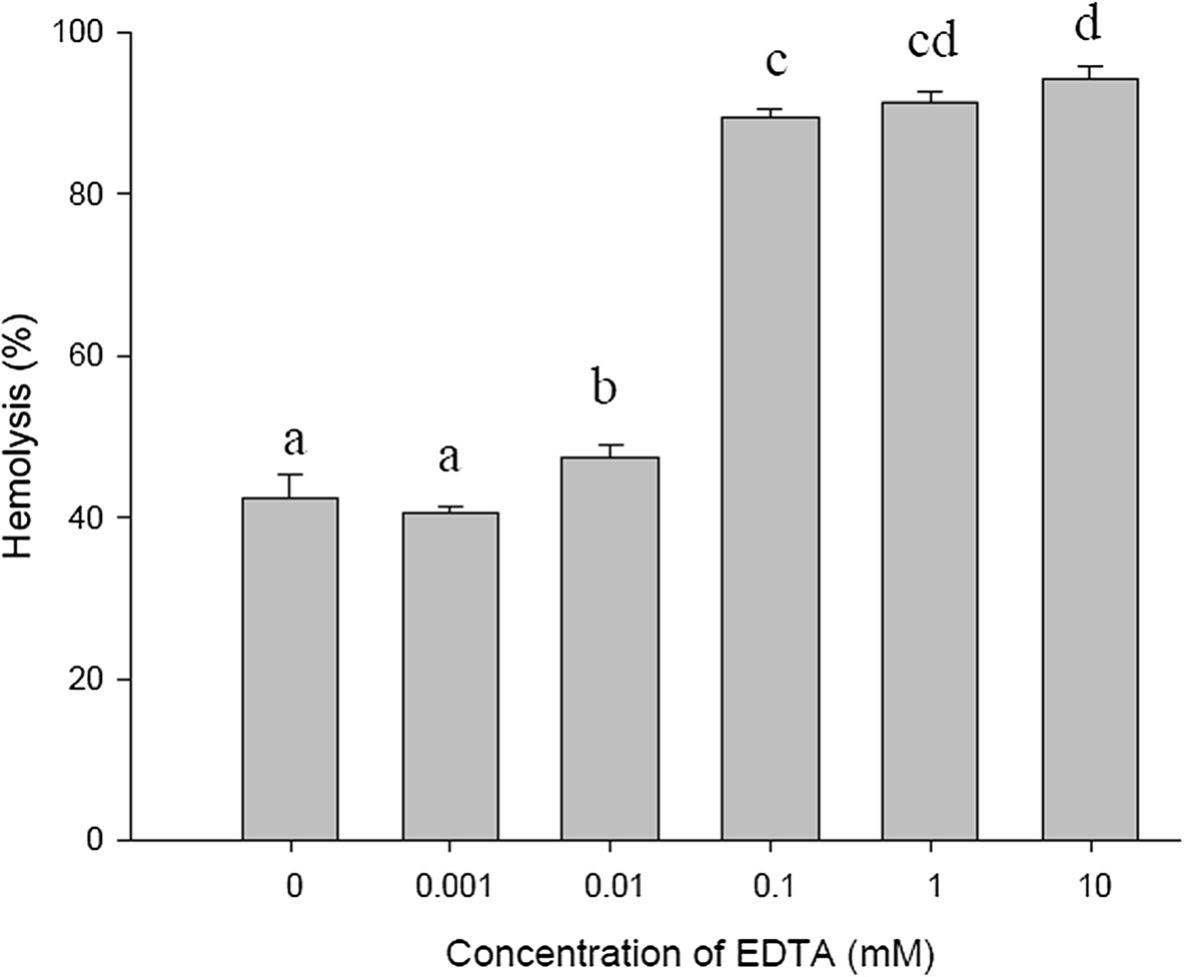
**Effect of EDTA on the hemolytic activity of the ASV solution (15 μg/mL).** Aliquots of ASV were added into a 0.5% erythrocyte suspension containing different EDTA concentrations. Values **a**, **b**, **c**, **d** differ significantly at *p* < 0.05 (n = 3).

Figure 4 demonstrates that the hemolytic activity of ASV was enhanced by the addition of EDTA at concentrations greater than 0.1 mM. Adding 10 mM EDTA to the hemolysis assay increased the hemolytic activity of ASV from 45.06 to 93.48%.

### Effect of cholesterin on the hemolytic activity of ASV

**Figure 5:**
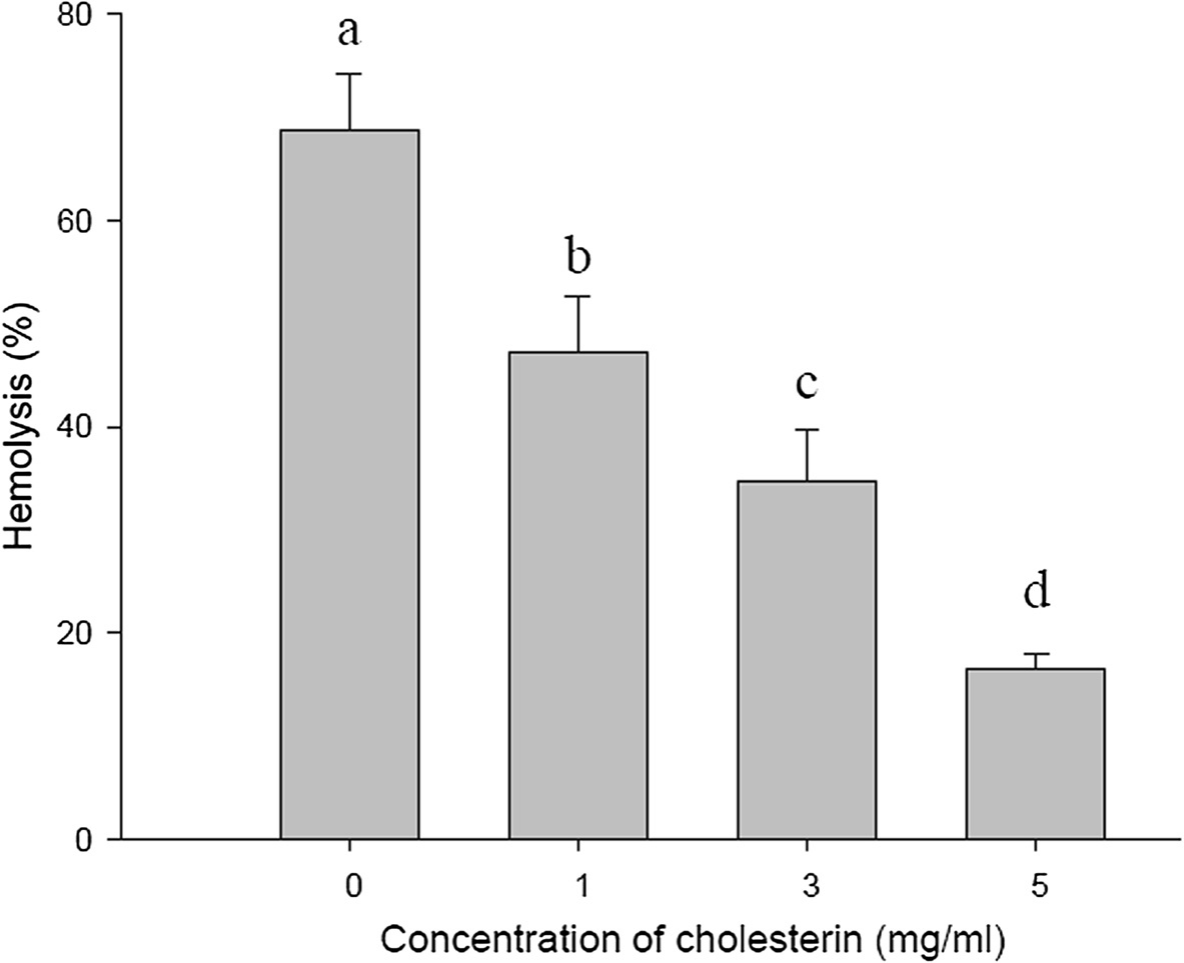
**Effect of cholesterin on the hemolytic activity of the ASV solution (20 μg/mL).** Aliquots of ASV were added into a 0.5% erythrocyte suspension containing different cholesterin concentrations. Values **a**, **b**, **c**, **d** differed significantly at *p* < 0.05 (n = 3).

To identify the membrane component responsible for cell sensitivity to ASV, the effect of membrane components, such as cholesterin and carbohydrates, on the hemolytic activity were tested. Carbohydrates did not affect the hemolysis (data not shown). Figure 5 shows that 1 mg/mL of cholesterin decreased the hemolytic activity of ASV from 68.74 to 47.26%. The presence of 5 mg/mL of cholesterin reduced the hemolytic activity by approximately 50%.

### Effect of proteases

**Figure 6:**
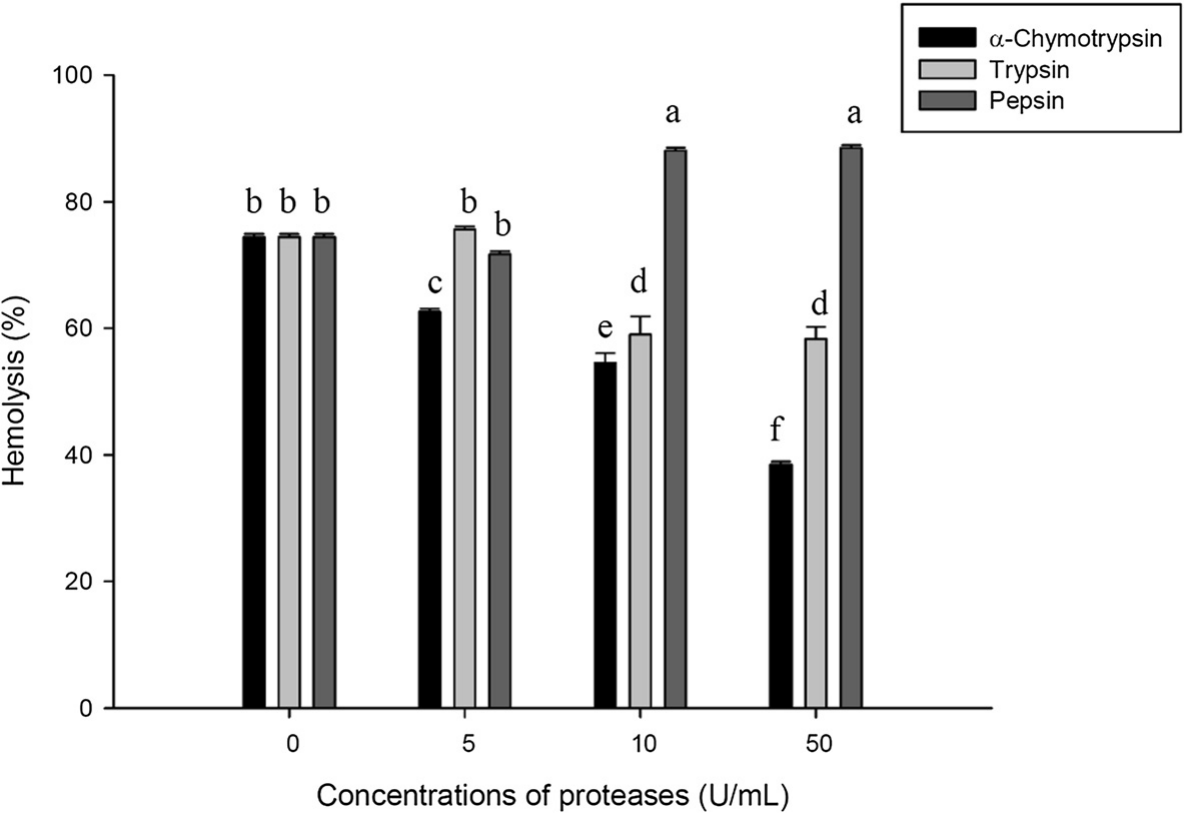
**Effect of proteases on the hemolytic activity of the ASV solution (20 μg/mL).** Aliquots of ASV were added into a 0.5% erythrocyte suspension containing different concentrations of proteases. Values **a**, **b**, **c**, **d**, **e**, **f** differed significantly at *p* < 0.05 (n = 3).

As shown in Figure 6, the hemolytic activity of ASV was altered by different proteases. Pepsin improved the hemolytic potency of ASV. After incubation of ASV with 10 U/mL pepsin, the hemolysis percentage increased from 71.72 to 88.12%, indicating that pepsin improved the hemolytic potency of ASV. Increasing the concentration of pepsin to 50 U/mL did not enhance the hemolytic activity. However, hemolysis was reduced when ASV was treated with trypsin and α-chymotrypsin; the inhibitory effect depended on the concentration of α-chymotrypsin and trypsin. Incubation of ASV with 10 U/mL and 50 U/mL of α-chymotrypsin reduced hemolysis by 20.15% and 36.09%, respectively. Incubation of ASV with 10 U/mL and 50 U/mL of trypsin reduced hemolysis by 17.32% and 18.17%, respectively.

### Effect of glycoside Hydrolases on the hemolytic activity of ASV

In our results, the effect of the glycoside hydrolases α-amylase and xylanase on the hemolytic activity of the venom was not significant. They affected the percentage of hemolysis by not more than 5% (data not show). Only cellulase produced a significant effect on the hemolytic activity of the venom.

**Figure 7:**
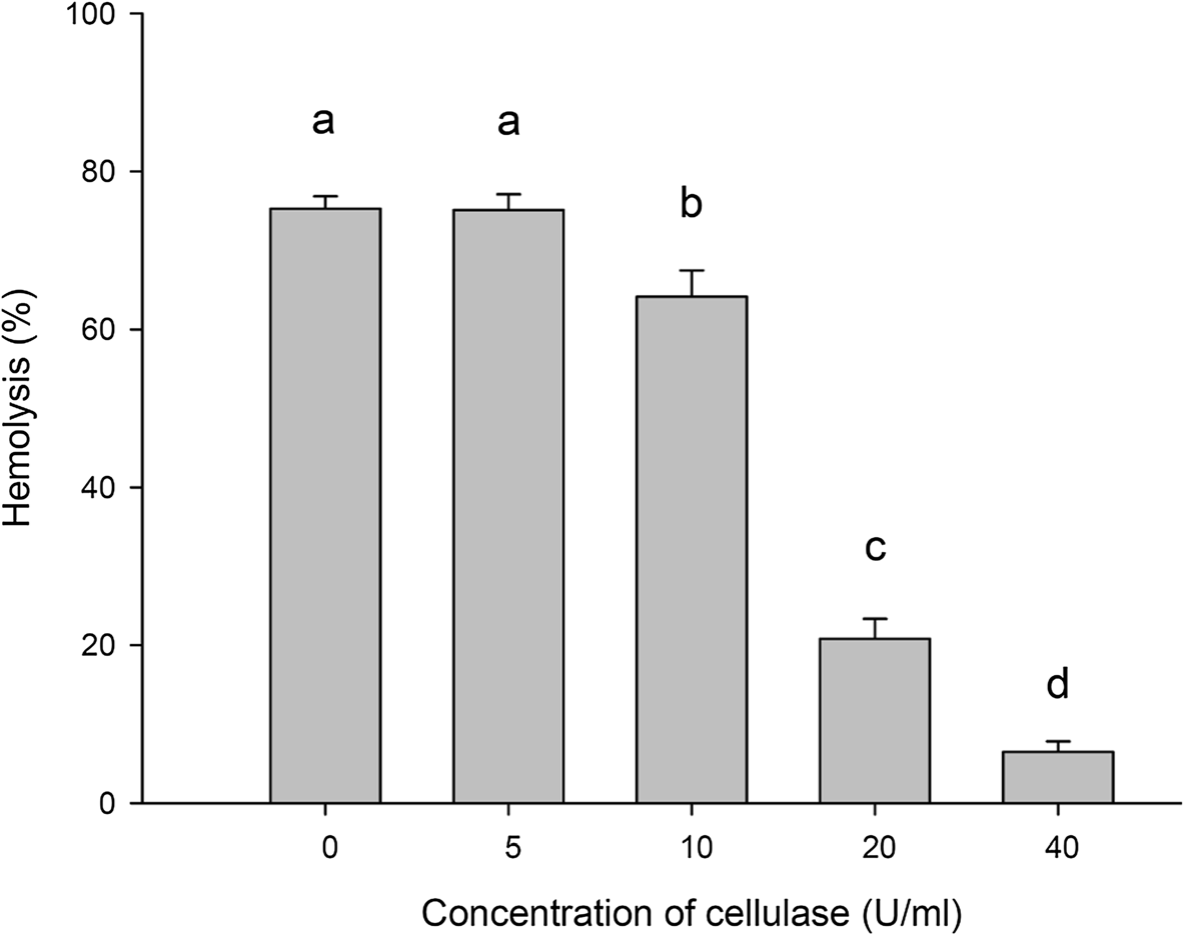
**Effect of cellulase on the hemolytic activity of the ASV solution (20 μg/mL).** Aliquots of ASV were added into a 0.5% erythrocyte suspension containing different cellulase concentrations. Values **a**, **b**, **c**, **d** differed significantly at *p* < 0.05 (n = 3).

As shown in Figure 7, the hemolytic activity of ASV was inhibited after cellulase treatment. The hemolytic activity decreased when ASV was treated with cellulase; the inhibitory effect depended on the concentration of cellulase. Incubating ASV with 20 U/mL and 40 U/mL of cellulase reduced hemolysis by 55.14 and 78.27%, respectively.

## Discussion

The crown-of-thorns starfish *A. planci* is a large Echinoderm whose surface is covered with spines. It can reach a diameter up to 50 cm, and the number of arms ranges from 10 to 20 [1,2].

In the present study, we focused on the *in vitro* characterization of the hemolytic activity of venom from the crown-of-thorns starfish *A. planci* spines. Our results showed that the venom from the spines of *A. palnci* from Taiwan exhibits strong hemolytic activity. The venom was characterized as a protein and such results are similar to previous reports [1-3].

PLA2s are enzymes that hydrolyze the sn-2 ester bond of phospholipids, and are abundant in the venoms of snakes and insects as well as in mammalian pancreatic juices. It is well known that PLA2s in snake venom are associated with various pathological symptoms in poisonings [9]. The hemolytic activity of the purified PLA2s from *A.planci* venom against sheep erythrocytes was assayed. In the presence of phosphatidylcholine (PC), PLA2s caused hemolysis at a much higher level than in the absence of PC. One study suggested that purified PLA2s from *A.planci* venom were very weak as to direct hemolysis, but exhibited indirect hemolytic activity [9]. In our study, we also assayed the hemolysis of ASV with and without egg yolk PC. However, in the presence of PC, ASV caused about 45% hemolysis at 0.25 μg/mL, and 100% hemolysis at 0.5 μg/mL (data not show). This result suggested that PLA2 plays an important role in the hemolysis of ASV.

In the present study, the hemolytic activity of ASV was preserved between pH 3.0 and 8.0 and was greatly inhibited at pH 2 and above pH 8. A previous study also reported that the venom from *A. planci* spines is unstable in acidic (pH < 3) or alkaline (pH > 10) environments [1]. Our results corroborated the instability of ASV hemolysis in an alkaline environment. These findings indicate that using ammonia diluted in water is useful for treating crown-of-thorns starfish envenomation.

Jellyfish toxins, which are similar to the venom from the crown-of-thorns starfish, have been characterized as proteinaceous [10,11]. The hemolytic activity caused by the venom from the jellyfish *Rhopilema esculentum* has been shown to be the most temperature-sensitive among all the jellyfish. When *R. esculentum* venom was incubated at temperatures over 40°C, its hemolytic activity was sharply reduced [12]. A previous study indicated that the venom from the crown-of-thorns starfish is a protein and loses its toxicity when heated to 60°C [1]. However, the present study showed that ASV was extremely stable over a wide range of temperatures. After incubation at 100°C for 30 minutes, ASV still exhibited 50% hemolytic activity. These findings indicate that heat treatment is not appropriate for reducing the effects of *A. planci* envenomation.

When examining the effect of different metal ions on the hemolytic activity of ASV, our study found that the heavy metal cation Cu^2+^ could alter the structure of the venom. Hemolytic activity was reduced in the presence of 1 mM of Cu^2+^. Sulfhydryl groups of cysteine residues in proteins react with Cu^2+^ to form undissolved mercaptans [13]. Therefore, protein activities probably become inhibited due to structural changes promoted by the onset of covalent bonds, which may account for the fact that Cu^2^ inhibits the hemolytic activity of ASV.

Ca^2+^ enhanced the hemolytic activity of ASV. In other studies, Ca^2+^ enhanced the hemolysis of venom from the jellyfish *Cyanea nozakii* and *Aiptasia pallida*[7,14]. The formation of pores in the membrane of erythrocytes induced by the jellyfish venom is dependent on the Ca^2+^ concentration of the medium, and most likely results from dimerization of the toxin within the membrane [15]. The hemolytic activity of ASV was enhanced by EDTA at a concentration 0.1 mM. Because EDTA is a chelator, it chelates heavy metal cations, such as Cu^2+^, thus inhibiting their effect on hemolytic activity [13].

Cholesterin, found in every cell, is especially abundant in cell membranes, whose integrity it helps maintain, and plays a role in facilitating cell signaling [16]. Cholesterin is thought to represent the binding receptor for the hemolytic venom. The hemolytic activity of ASV was significantly inhibited by cholesterin, a finding that indicates that the hemolytic toxin in ASV exhibited biological activity by recognizing cholesterin. Therefore, cholesterin most likely decreases the hemolysis-related lesion caused by ASV on the membranes of erythrocytes.

Trypsin was reported to inhibit hemolytic activities caused by the jellyfish *Cyanea nozakii* Kishinouye, which produces proteinaceous venom [7]. In the present study, treatment of ASV with α-chymotrypsin reduced the hemolytic activity. The effect was dose-dependent, and the trypsin treatment induced a slight decrease in the hemolytic activity. Therefore, α-chymotrypsin is most likely a useful protease for treating *A. planci* envenomation. However, treatment of ASV with pepsin induced a slight increase in the hemolytic activity; the hemolysis percentage increased from 71.71 to 88.52%. The hemolytic component in ASV is probably degraded by pepsin into subunits with a stronger hemolytic potency than the initial material.

Cellulose is a polysaccharide formed by the polymerization of a few hundred to over several thousand β-(1,4)-D-glucose units [17,18]. Cellulase can degrade native cellulose into soluble sugars [19]. In the present study, after treatment with cellulase, the hemolytic activity of ASV was inhibited by cellulose in a dose-dependent manner. This finding shows that the hemolytic components of ASV most likely contain glycosides, such as cellulose. Cellulase degraded the cellulose glycosides of the hemolytic components of ASV, which reduced the hemolytic activity of the venom.

## Conclusions

The hemolytic activity is affected by pH, temperature, metal ions, EDTA, cholesterin, proteases, and glycoside hydrolases. Given that Cu^2+^, cholesterin, α-chymotrypsin, and cellulase inhibit ASV hemolysis, these factors might be available to prevent the hemolytic activity of venom and provide the medical treatment for sting. ASV is unstable in an alkaline environment. Daubing the afflicted site with alkali might be employed to treat *A. planci* envenomation. ASV is extremely stable over a wide range of temperatures. Heat treatment is not appropriate for reducing the effects of *A. planci* envenomation. The results of this study provide fundamental information about *A. planci* spine venom. Further studies will differentiate and characterize the individual hemolytic components in *A. planci* spine venom and elucidate the action mechanisms of the venom.

## Competing interests

The authors declare that there are no competing interests.

## Authors’ contributions

CCL contributed to conception, design, acquisition, analysis and interpretation of data. WST was responsible for collecting samples. HJH was responsible for collecting samples. DFH drafted the article or revised it critically for important intellectual content. All authors read and approved the final manuscript.
